# P02.129. Individualized chiropractic and integrative care for low back pain: a randomized clinical trial

**DOI:** 10.1186/1472-6882-12-S1-P185

**Published:** 2012-06-12

**Authors:** G Bronfort, M Maiers, R Evans, K Westrom

**Affiliations:** 1 Northwestern Health Sciences University, Bloomington, USA

## Purpose

To determine the relative effectiveness of multidisciplinary integrative care compared to chiropractic care for chronic LBP in patients over 18 years of age using pain as the primary outcome measure. Secondary outcomes were patient self-reported disability, global perceived effect, general health status, satisfaction, self-efficacy, fear avoidance behavior, lumbar dynamic motion, and trunk muscle strength and endurance.

## Methods

Patients received 12 weeks of care with followup at 6 and 12 months. Both interventions had care teams who followed a care pathway designed to guide evidence based clinical decision making within the intervention groups. Patient profiles, based on a bio-psycho-social perspective, were created based on history, clinical examination findings, and patient preferences. Care teams met weekly to review patient profiles and make treatment recommendations based on patient presentation, the best available evidence, and their clinical experience. Integrative Care included acupuncture, chiropractic care, cognitive behavioral therapy, exercise, massage and medication in different combinations. Chiropractic care included spinal manipulation,selfcare advice, and home exercise.

## Results

Two hundred patients participated. Mixed model longitudinal analysis showed that the integrative care group had statistically significant more pain reduction, perceived global improvement and satisfaction with care in both the short- (up to 12 weeks) and long-term (through 52 weeks; p≤ 0.05). The group differences were relatively small.

## Conclusion

Integrative care for chronic low back pain resulted in slightly better short- and long-term outcomes than chiropractic care.

